# Factors related to job burnout among older nurses in Guizhou province, China

**DOI:** 10.7717/peerj.12333

**Published:** 2021-10-21

**Authors:** Hu Jiang, Nanqu Huang, Xue Jiang, Jianghong Yu, Yehong Zhou, Hengping Pu

**Affiliations:** 1 Nursing Department, The Third Affiliated Hospital of Zunyi Medical University (The First People’s Hospital of Zunyi), Zunyi, Guizhou, China; 2 Drug Clinical Trial Institution, The Third Affiliated Hospital of Zunyi Medical University (The First People’s Hospital of Zunyi), Zunyi, Guizhou, China

**Keywords:** Burnout, Job stress, Professional identity, Older nurses

## Abstract

**Background:**

The nursing workforce shortage has long been a global concern, and with the aging of nurses, this problem has become more prominent. Nursing is recognized as a high-stress occupation, and nurses experience high levels of job burnout, which reduces their professional identity. Older nurses are an indispensable talent force for nursing teams and are extremely important for the stability of nursing teams and improvement in nursing quality. Exploring the mental health and influencing factors of older nurses is very beneficial for the stability and development of nurse teams and patients’ clinical outcomes.

**Purpose:**

This study aimed to investigate the level of job burnout and its influencing factors among older nurses in Guizhou Province, China and confirm the correlations among job burnout, professional identity and stress level.

**Methods:**

From July to August 2019, 520 registered nurses aged over 40 years in Guizhou Province, China were surveyed through the Questionnaire Star platform. The questionnaire contained the following four parts: a general information questionnaire, the Maslach Burnout Inventory (MBI), a professional identity scale, and a job stressors scale.

**Results:**

The results showed that the job burnout score of the 520 older nurses was 55.44 ± 18.62, which was moderate. The level of job burnout was positively correlated with the level of nurse stress and negatively correlated with the level of professional identity, which was influenced by various personal and social factors.

**Conclusions:**

This study not only revealed that job burnout was still at a moderate level, but also revealed its current status and influencing factors among older nurses in China.

## Introduction

Currently, the shortage of nurses is a global problem and a major challenge in the field of health care (Nardi & Gyurko, 2013; Reinert, Bigelow & Kautz, 2012). Due to workforce shortages, increasing in chronic diseases and aging, nurses are under high work pressure. Such pressure has become an occupational risk for nurses (Gu, Tan & Zhao, 2019). With the development of industrial production and continuous improvement in social life, stress has increasingly become the focus of research. Nurses who work under high pressure for a long period experience adverse effects on their physical and mental health (Fernandes, Nitsche & Godoy, 2017). High-pressure nursing work leads to an increased willingness to leave, a lower willingness to stay, and a shortage of nurses, which is not conducive to the stability and healthy development of the nursing team (Labrague et al., 2017) and is harmful to the clinical outcome of patients (Hall et al., 2016; Saleh et al., 2014).

Job burnout (JB) refers to individuals’ severe and continuous nervousness at work, inability to achieve expected work outcomes, and dislike of work produced by apathy and indifference (Maslach, Schaufeli & Leiter, 2001). According to a meta-analysis (Salyers et al., 2017), severe job burnout among nurses can reduce the quality of nursing care, threaten the safety of patients’ lives, cause irreparable losses to patients and their families, and negatively affect the reputation of hospitals. In addition, job burnout reduces nurses’ work efficiency and increases their error rates and turnover rates (Basar & Basim, 2016). An international survey that enrolled 54,738 nurses from 646 hospitals in eight countries showed that nurse burnout is a global trend (Poghosyan, Aiken & Sloane, 2009).

The professional identity of nurses refers to their recognition of the goals, social values and other factors of the nursing occupation and affects their work enthusiasm and service quality (Fagermoen, 1997). A previous study showed that the shortage of nurses and growing professional identity crisis have become global problem (Clark, 2010). Studies have shown that correlations exist among nurses’ professional identity, job burnout and turnover intention (Kim, Lee & Lee, 2019; Mahoney et al., 2020).

Nurses’ professional identity and burnout are influenced by various factors, including personal and social factors, and age is an influential factor. Currently, the aging of the nursing labor force is a global trend (Wells & Norman, 2009). The older worker is one defined as 40 years of age or older (US Equal Employment Opportunity Commission, 2009). Older nurses are defined as those aged over 40 years (Cui, Yin & Fan, 2013; Van der Heijden et al., 2020). According to the latest research, in many countries, the number of nurses aged over 50 years accounts for more than 30% of all nurses in the country (Ryan et al., 2019). Previous studies have shown that age is related to professional burnout, and young nurses are more prone to occupational burnout; thus, young nurses have become the focus (Gómez-Urquiza et al., 2017; Yu et al., 2019). Existing studies confirmed that older nurses experience severe psychological problems (Collins-McNeil, Sharpe & Benbow, 2012). In addition, due to their age, nursing skills, knowledge levels and other factors, the work ability of older nurses in clinical nursing may also be low (Stimpfel et al., 2020), rendering job burnout more likely (Lammintakanen & Kivinen, 2012). Older nurses are an indispensable talent force for nursing teams and are extremely important for the stability of nursing teams and improvement in nursing quality. Exploring the mental health and influencing factors of older nurses is very beneficial for the stability and development of nurse teams and patients’ clinical outcomes. Many studies investigated older nurses in foreign countries, but very few studies were conducted in China; in addition, the professional burnout characteristics and influencing factors of elderly nurses are still unclear. Therefore, the purpose of this study is to analyze the job burnout level and its influencing factors among older nurses in China and confirm the correlations among job burnout, professional identity and stress level.

The research questions of this study are as follows: (1) Which is the job burnout level of older nurses? (2) What are the factors influencing job burnout among older nurses in China?

## Material and Methods

### Design and participants

This study was a descriptive survey study. We built an electronic questionnaire on a special questionnaire survey website. We distributed the questionnaire through the mobile WeChat application. Nurses aged over 40 years were the target population. The nurses could complete and directly submit the questionnaire after completing the questionnaire.

The inclusion criteria were as follows: (1) working as a nurse for more than 1 year, (2) provided informed consent and were willing to participate in this study. The exclusion criteria were as follows: (1) stopped working as a nurse at 6 or more months before the survey (*e.g.*, studying abroad, transferring nursing jobs), (2) diagnosed with a psychological disorder, or (3) diagnosed with a physical disorder, such as visceral function failure, malignant tumors.

### Instruments

The general information questionnaire was designed by the researchers and included items concerning gender, age, marital status, academic degree, working years, job title, income, employment method, number of children, number of night shifts, hospital level, etc.

The Maslach Burnout Inventory (MBI), which was developed by American psychologists Maslach & Jackson (1981), was used to evaluate the respondents’ emotional reaction to work pressure and attitudes and perceptions of personal work. A Chinese MBI scale, which was revised by Chen, Liu & Meng (2018), was used to measure the job burnout of older nurses. This scale includes 22 items, and the Cronbach’s *α* is 0.763. There are three dimensions in the scale, *i.e.,* emotional exhaustion (Cronbach’s *α* is 0.863), personification (Cronbach’s *α* is 0.718), and personal accomplishment (Cronbach’s *α* is 0.812), and the scores of the three dimensions are calculated independently. Job burnout is divided into 4 levels: zero burnout (job burnout scores on all 3 dimensions are below the severe threshold), mild burnout (scores equal to or above the severe threshold on one factor in the job burnout scale), moderate burnout (scores on one of the two dimensions are equal to or higher than the severe threshold), and severe burnout (scores on both dimensions are equal to or above the severe burnout threshold).

Job stressors was designed according to China’s national conditions by Li & Liu (2000). The job stressors scale includes 35 items, and the Cronbach’s *α* is 0.98. The scale includes the following five dimensions: nursing profession and work problems (Cronbach’s *α* is 0.95), time allocation and workload (Cronbach’s *α* is 0.83), working environment and equipment problems (Cronbach’s *α* is 0.92), patient care issues (Cronbach’s *α* is 0.94), and management and interpersonal problems (Cronbach’s *α* is 0.90). The responses were provided on a 4-point Likert scale. The higher the score, the higher the nursing work stress level.

The professional identity scale was developed by Liu, Hao & Liu (2011). The professional identity scale includes a total of 30 items. The questionnaire has good reliability and validity, with a Cronbach’s *α* of 0.938, and can be used to measure nurses’ professional identity. The scale is divided into the following five dimensions: professional identity evaluation (Cronbach’s *α* is 0.911), professional social support (Cronbach’s *α* is 0.824), social skills (Cronbach’s *α* is 0.763), coping with professional frustration (Cronbach’s *α* is 0.830) and professional self-reflection (Cronbach’s *α* is 0.720). The scale uses a 5-point Likert scale. The questions are positively worded, and the total possible score is 150 points, with higher scores indicating higher professional identity levels. Scores of 30–60, 61–90, 91–120, and 121–150 were used to create the lower-, low-, medium-, and high-scoring groups, respectively.

### Procedures

The questionnaire was administered *via* Questionnaire Star. After obtaining written informed consent from the study subjects, the researchers presented unified instructions for completing the questionnaire and indicated matters needing attention to the respondents. The respondents could access the questionnaire online using the provided links or by scanning the provided QR code and submit it by clicking “submit” after completion. From July to August 2019, nurses aged over 40 years in Guizhou Province, China assessed their job burnout, professional identity and job stressors through the Questionnaire Star platform.

### Ethical considerations

This study was approved by the Ethics Committee of the First People’s Hospital of Zunyi (2019-061), and permission for the data collection was obtained from the participants. All participants received a written notification before participating in the study. The online questionnaire explained that the privacy of the participants would be fully protected and that all submitted information would only be used for research purposes.

### Statistical analysis

In this study, 521 questionnaires were collected through the Questionnaire Star platform; of these questionnaires, 520 were valid, yielding an effective recovery rate of 99.8%. One questionnaire was excluded because of a logical error in the information. Excel 2010 was used to build the database, however the SPSS 18.0 software package was used for the data analysis. The measurement data were presented as the mean ± standard deviation ($\overline{x}$ ±*s*). A Pearson correlation analysis was used to analyze the correlations among job burnout, job identity and job stress, and a one-way ANOVA, independent-samples *t-* test and multiple stepwise linear regression analysis were used to analyze the factors influencing job burnout. The effect size and 95% confidence intervals were calculated by one-way ANOVA and independent-samples *t-* test. The effect size was used to measure the magnitude of the difference. In the *t-* test, Cohen’s *d* value was used to indicate the effect size, and we considered *d* = 0.20 small, *d* = 0.50 medium, and *d* = 0.80 large effect sizes. Cohen’s *f* value was used to indicate the effect size of the ANOVA, and we considered *d* = 0.10 small, *d* = 0.25 medium, and *d* = 0.40 large effect sizes. The critical points of the effect size are 0.20, 0.50, and 0.80. Comparisons between the groups were performed by analyzing the data using the least significant difference (LSD) post hoc method. *P* < 0.05 was considered indicative of statistical significance.

## Results

### Basic information of the respondents

The basic information of the respondents is shown in Table 1. In total, 520 respondents aged 40–60 (45.89 ± 4.64) years, including 9 males (1.7%) and 511 females (98.3%), participated in this study; the average number of working years was 25.16 ± 5.35 years. In total, 84.3% of the nurses had intermediate or above professional titles. In total, 79.2% of the nurses had one child, 17.3% of the nurses had two or more children, and 73.1% of the nurses did not work night shifts. In total, 4.2% of the nurses were from primary hospitals, 49% of the nurses were from secondary hospitals and 46.7% of the nurses were from tertiary hospitals.

**Table 1:** Single-factor analysis of job burnout (*N* = 520).

Variable	N (%)	Burnout score	*t/F*	*P*	Effect size (95% *CI*)
Age (year)					
40–45	277 (53.3)	57.51 ± 18.76	4.211	0.006	0.156 (0.091∼0.275)
46–50	156 (30)	55.10 ± 18.93			
51–55	79 (15.2)	49.56 ± 16.89			
≥56	8 (1.5)	48.63 ± 13.58			
Gender					
Male	9 (1.7)	69.78 ± 18.33	5.464^a^	0.020	0.786 (0.583∼0.924)
Female	511 (98.3)	55.19 ± 18.56			
Job title					
Nurse	34 (6.5)	68.26 ± 18.14	7.948	0.000	0.248 (0.113∼0.438)
Senior nurse	100 (19.2)	59.67 ± 19.69			
Supervisor nurse	218 (41.9)	54.48 ± 17.50			
Cochief superintendent nurse	154 (29.6)	51.11 ± 18.01			
Chief superintendent nurse	14 (2.7)	56.71 ± 18.55			
Department					
Internal medicine	142 (26)	55.48 ± 19.49	2.368	0.013	0.204 (0.073∼0.482)
Surgery department	83 (17.7)	59.05 ± 19.14			
Gynecology and obstetrics	48 (9.6)	51.54 ± 19.23			
Pediatrics	46 (8.8)	61.41 ± 17.67			
Emergency department/intensive care unit	33 (6.2)	61.15 ± 16.85			
Radiological/imaging center	8 (1.9)	47.88 ± 21.21			
Outpatient service	35 (5.2)	54.63 ± 17.49			
Operating room/sterile supply room	56 (11)	53.07 ± 17.14			
Nursing department	40 (7.5)	49.53 ± 16.27			
Others	29 (2.5)	51.24 ± 17.89			
Working years (years)					
16–20	131 (25.2)	63.20 ± 17.65	10.069	0.000	0.280 (0.139∼0.497)
21–25	168 (32.3)	53.67 ± 18.41			
26–30	147 (28.3)	54.50 ± 19.44			
31–35	56 (10.8)	47.84 ± 15.17			
≥36	18 (3.5)	46.94 ± 11.63			
Income (CNY)					
3000–5999	191 (36.7)	59.32 ± 18.83	6.141^b^	0.000	0.189 (0.089∼0.384)
6000–8999	244 (46.9)	54.51 ± 18.56			
9000–11999	67 (12.9)	49.09 ± 16.75			
≥12000	18 (3.5)	50.56 ± 16.34			
Number of children					
0	18 (3.5)	69.11 ± 16.31	12.399^b^	0.000	0.268 (0.097∼0.528)
1	412 (79.2)	53.04 ± 17.69			
2	86 (16.5)	63.40 ± 19.81			
>2	4 (0.8)	70.75 ± 23.66			
Hospital grade					
Primary hospital	22 (4.2)	57.73 ± 21.04	3.112^b^	0.045	0.110 (0.028∼0.385)
Secondary hospital	255 (49)	57.31 ± 18.87			
Tertiary hospital	243 (46.7)	53.28 ± 18.00			

**Notes.**

a *t* value.

b *n* equal variance not assumed.

### Single-factor analysis of the job burnout of older nurses

The single-factor analysis and effect size are shown in Table 1. The ANOVA of job burnout revealed a significant difference among the older nurses by age (*F* = 4.211, *P* = 0.006). The LSD post hoc analysis showed that older nurses aged 51–55 years had a lower level of job burnout than those aged 40–45 years (*P* = 0.001) and 46-50 years (*P* = 0.030). The independent-samples *t-* tests showed a significant difference between the genders (*t* = 5.464, *P* = 0.020), males had a higher level of job burnout. The ANOVA of job burnout revealed a significant difference among the older nurses by job title (*F* = 7.948, *P* = 0.000). The LSD post hoc analysis showed that older nurses with a nurse job title had higher job burnout than those with a job title of senior nurse (*P* = 0.017), supervisor nurse (*P* = 0.000), cochief superintendent nurse (*P* = 0.000), and chief superintendent nurse (*P* = 0.046). The ANOVA showed a significant difference among the older nurses by department (*F* = 2.368, *P* = 0.013). The ANOVA showed that job burnout significantly varies by the length of work years (*F* = 10.069, *P* = 0.000). The LSD post hoc analysis showed that the nurses who worked for 16–20 years had higher job burnout than those who worked for 21–25 years (*P* = 0.000), 26–30 years (*P* = 0.000), 31–35 years (*P* = 0.000), and ≥36 years (*P* = 0.000). There was a significant difference among the different income groups (*F* = 6.141, *P* = 0.000). The LSD post hoc analysis showed that the lower income groups had higher job burnout, especially between the 3000–5999 group and the 6000–8999 group (*P* = 0.007) and 9000-11999 group (*P* = 0.000). The ANOVA showed that job burnout significantly varied by the number of children (*F* = 12.399, *P* = 0.000), and the LSD post hoc analysis showed that older nurses with one child had lower job burnout than those who had no children (*P* = 0.000) and 2 children (*P* = 0.000). Our results showed that nurses’ job burnout differed by hospital grade (*F* = 3.112, *P* = 0.045), and the LSD post hoc analysis revealed that secondary hospitals had more serious burnout than tertiary hospitals (*P* = 0.016).

### Job burnout, job stressors and professional identity scores

The results showed that the job burnout scores of the respondents were as follows: the average emotional exhaustion dimension score was 26.58 ± 11.54, with an average item score of 2.95 ± 1.28; the average personification dimension score was 10.42 ± 6.13, with an average item score of 2.08 ± 1.23; and the average personal accomplishment dimension score was 18.44 ± 8.45, with an average item score of 2.30 ± 1.06. The job stressor and professional identity scores of the nurses are shown in Table 2.

### Pearson correlation analysis of job burnout, job stressors and professional identity

The Pearson correlation analysis results showed that the job stressor scores were positively correlated with the total job burnout score (*r* = 0.477) and that the job stressor scores were positively correlated with the emotional exhaustion (*r* = 0.527) and personification (*r* = 0.443) scores. Nursing profession and work problems were positively correlated with emotional exhaustion (*r* = 0.371) and the total job burnout score (*r* = 0.341). Time allocation and workload were positively correlated with emotional exhaustion (*r* = 0.463), personification (*r* = 0.326), and the total job burnout score (*r* = 0.380). The working environment and equipment problems were positively correlated with emotional exhaustion (*r* = 0.437), personification (*r* = 0.374), and the total job burnout score (*r* = 0.398). Patient care issues were positively correlated with emotional exhaustion (*r* = 0.476), personification (*r* = 0.438), and the total job burnout score (*r* = 0.419). Management and interpersonal problems were positively correlated with emotional exhaustion (*r* = 0.487), personification (*r* = 0.423), and the total job burnout score (*r* = 0.471). However, these five dimensions were not correlated with personal accomplishment.

**Table 2:** Job burnout, job stressors and professional identity scores of older nurses (*n* = 520, $\bar {x}~\pm $ s).

Variable	Score	Average score of the dimension items
Job burnout	55.44 ± 18.62	
Emotional exhaustion	26.58 ± 11.54	2.95 ± 1.28
Personification	10.42 ± 6.13	2.95 ± 1.28
Personal accomplishment	18.44 ± 8.45	2.30 ± 1.06
Job stressors	18.44 ± 8.45	
Professional identity	114.90 ± 20.17	

The total professional identity scores were negatively correlated with personal accomplishment (*r* =  − 0.564) and the total job burnout score (*r* =  − 0.398). Professional identity evaluation was negatively correlated with personal accomplishment (*r* =  − 0.506) and the total job burnout score (*r* =  − 0.410). Professional social support was negatively correlated with personal accomplishment (*r* =  − 0.501) and the total job burnout score (*r* =  − 0.337). Social skills were negatively correlated with personal accomplishment (*r* =  − 0.509). Professional frustration was negatively correlated with personal accomplishment (*r* =  − 0.563) and the total job burnout score (*r* =  − 0.397). Professional self-reflection was negatively correlated with personal accomplishment (*r* =  − 0.479) and the total job burnout score (*r* =  − 0.354). No dimension of professional identity was correlated with emotional exhaustion and personification. The results are shown in Table 3.

**Table 3:** Correlation analysis of job burnout, job stressors and professional identity of nurses (*r* value).

Variable	Emotional exhaustion	Personification	Personal accomplishment	Job burnout total score
Job stressors				
Nursing profession and work problems	0.371^**^	0.288^**^	0.037^**^	0.341^**^
Time allocation and workload	0.463^**^	0.326^**^	−0.030^**^	0.380^**^
Working environment and equipment problems	0.437^**^	0.374^**^	0.009^**^	0.398^**^
Patient care issues	0.476^**^	0.438^**^	−0.042 ^**^	0.419^**^
Management and interpersonal problems	0.487^**^	0.423^**^	0.068^**^	0.471^**^
Job stressor total score	0.527^**^	0.443^**^	0.012^**^	0.477^**^
Professional identity				
Professional identity evaluation	−0.257^**^	−0.064^**^	−0.506^**^	−0.410^**^
Professional social support	−0.144^**^	−0.064^**^	−0.501^**^	−0.337^**^
Social skills	−0.096^**^	0.034^**^	−0.509^**^	−0.279^**^
Professional frustration	−0.180^**^	−0.093^**^	−0.563^**^	−0.397^**^
Professional self-reflection	−0.183^**^	−0.073^**^	−0.479^**^	−0.354^**^
Professional identity total score	−0.201^**^	−0.056^**^	−0.564^**^	−0.398^**^

**Notes.**

** Statistically significant difference between the groups **P < 0.001

### Multiple linear regression analysis of job burnout

Personal characteristics, job stressors, and professional identity were used as independent variables, and job burnout was used as a dependent variable in the multiple linear regression analysis. The goodness of fit of the model was 0.447, indicating that the degree of interpretation of the data information by this equation reached 44.7%. The corresponding *P*-value was 0.000, which is less than 0.05, indicating that the model was reasonable. The results showed that working years, number of children, hospital grade, nursing profession and work problems, time allocation and workload, patient care issues, management and interpersonal problems, professional identity evaluation, and professional frustration were significantly related to job burnout (*P* < 0.05). The results are shown in Table 4.

**Table 4:** Multiple linear regression analysis of job burnout.

Variables	*B*	*SE*	*β*	*t*	*P*
Constant term	71.350	13.360	–	5.341	0.000
Working years	−2.382	1.047	−0.137	−2.274	0.023
Number of children	3.735	1.460	0.092	2.559	0.011
Hospital grade	3.000	1.325	0.092	2.265	0.024
Nursing profession and work problems	−0.603	0.198	−0.171	−3.046	0.002
Time allocation and workload	0.655	0.259	0.128	2.528	0.012
Patient care issues	0.413	0.155	0.148	2.662	0.008
Management and interpersonal problems	0.768	0.178	0.252	4.320	0.000
Professional identity evaluation	−0.571	0.161	−0.227	−3.537	0.000
Professional frustration	−1.188	0.387	−0.265	−3.072	0.002

**Notes.**

*F* = 16.700, *P* = 0.000, *R*^2^ = 0.447, adjusted *R*^2^ = 0.421.

## Discussion

In this study, we found that the burnout level of older nurses was positively correlated with their stress levels and negatively correlated with their levels of professional identity. Job burnout was affected by various factors. The lower the nurses’ age, working years, professional title and salary level, the higher their job burnout scores.

The mutual mechanism influencing nurses’ stress levels, job burnout, professional identity and turnover rate has been gradually understood. Some conceptual models suggest that nurse stress and job burnout are related through a cause-and-effect relationship and that the burnout level affects organizational commitment and professional identity, while the latter two variables further influence each other (Chen & Chen, 2018). In this study, although the nurses’ total job stressor score was positively correlated with their job burnout, the time allocation and workload score and patient care issues score were negatively correlated with personal accomplishment, further showing that the greater the workload, the higher the nurses’ stress levels, which significantly affected their personal achievement. In long-term care for patients, increased workload and working hours cause nurses to suffer from stress and anxiety, and in the long run, their physical and mental health are damaged, forming a vicious circle (Chen et al., 2020; Priano, Hong & Chen, 2018). Therefore, long-term pressure may be an important factor causing job burnout among nurses. Timely and correct treatment can slow or prevent the decline in professional identity and turnover (Aryankhesal et al., 2019).

In this study, the nurses’ emotional exhaustion (26.58 ±11.54) and personal accomplishment (18.44 ± 8.45) dimension scores were at a high level, and their personification (10.42 ±6.13) dimension score was at a medium level. Regarding other research concerning this topic in China, the burnout level in our study was higher than that reported in Wu’s research (Wu et al., 2014), while the personal accomplishment scores in this study were lower than those reported by Yang et al. (2018), and the other two dimension scores were higher. This difference may have been due to the region studied and the samples used. This study further confirmed that the level of job burnout of nurses was affected by age, working years, professional title, salary level, heavy workload, and number of children, which is consistent with the conclusions of other studies (Chen & Chen, 2018; Maruyama, Suzuki & Takayama, 2016).

In our study, male nurses had a higher level of job burnout than female nurses, even though with the limitation of samples. Researchers proved that gender was being male were related to the highest levels of burnout (Ortega et al., 2018). Currently, male nurses are the minority in China. However, Xian et al. (2020) proved that the job burnout of male nurses is at a serious situation. Studies concerning the mental status of male nurses aged over 40 years are still lacking, and more research is needed to reveal this practical situation.

Age is an important risk factor for job burnout. In this study, the job burnout level among the nurses aged 40–50 was significantly higher than that among the nurses aged 51–55, and the nurses aged 40–45 had the highest job burnout level, which is similar to previous studies (Membrive-Jiménez et al., 2020). Many studies have shown that nurses with low seniority have a higher level of job burnout (Chen & Chen, 2018; Wu et al., 2014; Yang et al., 2018), and this association between age and job burnout may be due to nurses’ work experience. Working years are related to nurses’ work experience, and most older nurses have worked for more than 20 years. Our study found that nurses aged 40–45 who worked for 16–20 years had the highest job burnout level, which may be caused by many reasons, such as family-work conflict and high-intensity work pressure (Fang, 2017; Klein et al., 2020), while older nurses have more experience coping with stress to reduce burnout (Kshetrimayum, Bennadi & Siluvai, 2019).

Older nurses constitute a group that merits concern worldwide. Due to the reduced number of young nurses, the proportion of older nurses is gradually increasing (Wells & White, 2014). This phenomenon is particularly notable in the United States, where it is estimated that half of nurses will reach the age of 65 in 2020 (Harrington & Heidkamp, 2013) and that 1 million nurses will retire by 2030 (Sofer, 2018). However, the proportion of older nurses in China is not quite the same as that in other countries, and the nursing workforce in China is still mainly younger, resulting in the neglect of older nurses. However, the younger nursing workforce in China is one of the notable features of the current composition of nurses (Kalisch & Liu, 2009). The proportion of nurses aged over 35 is not large, accounting for only 23% of the nursing workforce, while the proportion of nurses under 35 accounts for 77.0% (Xu et al., 2016).

Work overload is significantly associated with burnout among nurses, especially emotional exhaustion (Sadati et al., 2017). Nurses’ job burnout is correlated with patients’ care quality (Koy et al., 2015), and high nurse burnout is associated with increased odds of reporting negative patient outcomes, such as falls, medication errors, infections, and nursing tasks left undone, leading to lower ratings of the quality of care (Nantsupawat et al., 2016; Poghosyan et al., 2010; White, Aiken & McHugh, 2019). In addition, high nurse burnout is associated with lower levels of patient satisfaction (White, Aiken & McHugh, 2019). Although the burnout level of young nurses is higher than that of older nurses, the burnout level of older nurses is also at a high level compared with that of other professionals (Collins-McNeil, Sharpe & Benbow, 2012), which is worthy of attention. Older nurses have rich life experiences and work skills, excellent communication skills, dedication and loyalty and can mentor young nurses to develop (Uthaman, Chua & Ang, 2016). Without attention to these nurses’ burnout levels, the loss of older nurses will have an even greater impact on the health sector.

Although our study has increased the understanding of the current level of job burnout and professional identity of older nurses in China, there were still some limitations to our study. First, only nurses from Guizhou Province were recruited, and male nurses recruited in our studies were limited, limiting the generalizability of the findings. Second, the research method was an online questionnaire survey, and we could not determine the authenticity of the respondents’ responses to the questionnaire. The response rate of older nurses approaching retirement age was low; thus, the survey results may not be completely reliable, and further research is needed. Third, the survey participants were nurses aged over 40 years, which slightly differs from the definition of older nurses in other studies. In addition, the results obtained in this study were not compared with younger age groups aged less than 40 years. The results only represent the situation in this region, and more comparative studies are needed.

## Conclusions

In summary, older nurses were facing a moderate level of job burnout. This study showed that the burnout levels of older nurses were positively correlated with their stress levels and negatively correlated with their levels of professional identity, which were influenced by various personal and social factors such as working years, number of children, hospital grade and stress level etc. Therefore, it is necessary to focus on physical and mental pressure of older nurses and ensure health to maximize the effectiveness of the whole nursing team.

## Supplemental Information

10.7717/peerj.12333/supp-1 Supplemental Information 1 Raw data of Tables 1–4

10.7717/peerj.12333/supp-2 Supplemental Information 2 Blank copy of the questionnaire in Chinese

## Additional Information and Declarations

### Data Availability

The following information was supplied regarding data availability: The raw measurements are available in the Supplemental File.
