# Supplementary material for: Factors related to job burnout among older nurses in Guizhou province, China
Source: PeerJ. 2021 Oct 21;9:e12333. doi: 10.7717/peerj.12333 (PMC8542368; doi:10.7717/peerj.12333)
Supplement: Supplemental Information 2 [file peerj-09-12333-s002.docx]

高龄护士岗位任职现状与职业认同职业倦怠相关性研究调查问卷

**知情同意书**

我们诚挚邀请您参与此项目研究，这份知情同意书提供您了解关于本研究的信息、您需要遵照的事项以及本研究的相关风险与收益。请仔细阅读，在充分理解本研究相关内容的基础上做出是否参与的决定。您的决定是完全自主的。

**研究目的**：分析高龄护士的职业倦怠，职业认同和压力水平。

调查程序：此调查表将花费您大约10分钟。调查内容包括与职业倦怠，职业身份和压力源有关的问题。所有问题都没有固定答案，您可以根据自己的情况选择。本研究只关注您个人的意见。

**收益：**您的参与将有助于我们更好地了解高龄护士的身心健康，并获得社会和上级的关心和关注。此外，研究结果将有助于护理决策者。

**隐私与保密措施：**数据收集过程完全是匿名的。该数据不包含任何个人身份信息。收集的数据仅用于护理研究，除研究人员外，信息严格保密。

**权利：**您有权决定是否参与，并在任何时候终止参与而不会因此受任何不良待遇。

**您是否愿意参加：□是□否**

一、基本情况

1. 您的年龄：
2. 您的性别：男 女
3. 您的最高学历： ①中专 ②大专 ③本科 ④硕士 ⑤博士
4. 您现在的职称是：①护士 ②护师 ③主管护师 ④副主任护师 ⑤主任护师
5. 您目前的职务是:①责任组长/副护士长 ②护士长 ③护理部副主任/主任

④副院长/院长 ⑤其他 ⑥无职务

1. 您所在的科室是：
2. 您的工作年限：
3. 您的平均月收入：3000～5999 6000～8999 9000～11999 12000以上
4. 您的任职方式是：编制 合同 临聘
5. 您的婚姻状况是：①已婚 ②未婚 ③丧偶 ④离异
6. 您的子女个数：无 1个 2个 2个以上
7. 您是否为专科护士：是 否
8. 您每月的晚夜班数量是：0个 1个 2个 3个 3个以上

14.您工作的医院的等级为:一级医院 二级医院 三级医院

二、中国护士工作压力源量表

|  | 内容 | 非常不同意 | 不同意 | 同意 | 非常同意 |
| --- | --- | --- | --- | --- | --- |
| 护理  专业  及工  作问  题 | 护理工作的社会地位太低 |  |  |  |  |
|  | 继续深造的机会太少 |  |  |  |  |
|  | 工资及其他福利待遇低 |  |  |  |  |
|  | 晋升的机会太少 |  |  |  |  |
|  | 需要经常倒班 |  |  |  |  |
|  | 工作中的独立性太少 |  |  |  |  |
|  | 工作分工不明确 |  |  |  |  |
| 工作  量及  时间  分配  问题 | 工作量太大 |  |  |  |  |
|  | 上班护士数量少 |  |  |  |  |
|  | 没有时间对病人实施心理护理 |  |  |  |  |
|  | 非护理性的工作太多 |  |  |  |  |
|  | 无用的书面工作太多 |  |  |  |  |
| 工作环境及资源方面问题 | 工作环境太差 |  |  |  |  |
|  | 工作中所需要的仪器设备不足 |  |  |  |  |
|  | 病区拥挤嘈杂 |  |  |  |  |
| 病人护理方面问题 | 担心工作中出现差错事故 |  |  |  |  |
|  | 护士工作未被患者及家属承认 |  |  |  |  |
|  | 患者病情过重 |  |  |  |  |
|  | 患者的家属不礼貌 |  |  |  |  |
|  | 患者的要求太高或者太过分 |  |  |  |  |
|  | 患者不礼貌 |  |  |  |  |
|  | 患者不合作 |  |  |  |  |
|  | 所学的知识不能满足患者及家属的心理需求 |  |  |  |  |
|  | 缺乏患者教育的有关知识 |  |  |  |  |
|  | 担心护理操作会引起患者的疼痛 |  |  |  |  |
|  | 护理的患者突然死亡 |  |  |  |  |
| 管理及人际方面问题 | 缺乏其他卫生工作人员的理解及尊重 |  |  |  |  |
|  | 护理管理者的理解与支持不够 |  |  |  |  |
|  | 护理管理者的批评过多 |  |  |  |  |
|  | 医生对护理工作过分挑剔 |  |  |  |  |
|  | 同事之间缺乏理解与支持 |  |  |  |  |
|  | 与护理管理者发生冲突 |  |  |  |  |
|  | 与病区的某些护士工作很难 |  |  |  |  |
|  | 与医生发生冲突 |  |  |  |  |
|  | 同事之间缺乏友好合作的气氛 |  |  |  |  |

三、护士职业认同问卷

| 编号 | 条目 | 非常不符合 | 不太符合 | 不确定 | 比较符合 | 非常符合 |
| --- | --- | --- | --- | --- | --- | --- |
| 1 | 护理工作让我有价值感 |  |  |  |  |  |
| 2 | 我觉得有把握和我遇到的任何一个人接近并打交道 |  |  |  |  |  |
| 3 | 同事的理解与支持可使我获得幸福感 |  |  |  |  |  |
| 4 | 我深信人有主体选择性和能动性，不仅能适应职业环境，还能创造和优化职业环境 |  |  |  |  |  |
| 5 | 我认为择业既有必然性也有偶然性，你认可他，才会有所作为。 |  |  |  |  |  |
| 6 | 护理工作与我的兴趣，性格相匹配。 |  |  |  |  |  |
| 7 | 护理职业可使我更容易赢得社会大众的尊重 |  |  |  |  |  |
| 8 | 我喜欢与人交流，热衷于寻找社交接触的机会 |  |  |  |  |  |
| 9 | 医生和管理者的认可使我获得幸福感 |  |  |  |  |  |
| 10 | 我感到个体可在职业生涯中得到锻炼和成长 |  |  |  |  |  |
| 11 | 我认为专心在自己所从事职业的人，一定能从职业生涯中获得丰厚的回馈 |  |  |  |  |  |
| 12 | 离开护士职业会给我造成一些情感创伤 |  |  |  |  |  |
| 13 | 我每天的工作中充满了令我感兴趣的事情 |  |  |  |  |  |
| 14 | 我对自己的社交举止很自信 |  |  |  |  |  |
| 15 | 护理工作可使我和我的家人握有宝贵的医疗资源 |  |  |  |  |  |
| 16 | 职业生涯中遇到困难时，我会积极正面思考，努力寻找解决方法，不轻言放弃。 |  |  |  |  |  |
| 17 | 我觉得常分析自己的优势和劣势，可以缩小职业生涯中理想自我和现实自我的差距 |  |  |  |  |  |
| 18 | 从事护理职业令我愉快 |  |  |  |  |  |
| 19 | 面对一个难题时，我通常能找到几个解决方法 |  |  |  |  |  |
| 20 | 患者和家属的满意评价可使我获得幸福感 |  |  |  |  |  |
| 21 | 职业生涯中遇到挫折时，我不把它当绊脚石，而把它当垫脚石，将其视为攀登更高目标的磨练。 |  |  |  |  |  |
| 22 | 护理工作可使我施展个人的能力和特长 |  |  |  |  |  |
| 23 | 我更愿和强过自己的人比较，以此激励自己 |  |  |  |  |  |
| 24 | 心情不好时，我会找好友倾述，听音乐或参加体育活动等调整自己 |  |  |  |  |  |
| 25 | 如果我尽力去做，我总是能解决问题 |  |  |  |  |  |
| 26 | 目前岗位可使我发挥自身能力，获得幸福感 |  |  |  |  |  |
| 27 | 即使别人反对我，我任有办法取得我所要的 |  |  |  |  |  |
| 28 | 遇到困难时，我会与处境比我更困难的人比较 |  |  |  |  |  |
| 29 | 我有明确的职业发展目标 |  |  |  |  |  |
| 30 | 我对继续从事护理职业有种责任感 |  |  |  |  |  |

四、护士职业倦怠量表

|  | 内容 | 从来没有 | 一年几次 | 每月一次 | 每月几次 | 一周几次 | 每天都有 |
| --- | --- | --- | --- | --- | --- | --- | --- |
| 1 | 工作有时使我情绪低落 |  |  |  |  |  |  |
| 2 | 工作一天，我感到十分疲惫 |  |  |  |  |  |  |
| 3 | 早上起床时，我感到很疲乏，但仍然要面对当天工作 |  |  |  |  |  |  |
| 4 | 我能够设身处地的体会病人的感受 |  |  |  |  |  |  |
| 5 | 我有时感觉面对病人就像面对一件物品一样 |  |  |  |  |  |  |
| 6 | 整天与人打交道的工作，令我感到十分疲劳 |  |  |  |  |  |  |
| 7 | 我能够有效的处理病人护理过程中的各种问题 |  |  |  |  |  |  |
| 8 | 工作使我耗尽了心力 |  |  |  |  |  |  |
| 9 | 我觉得自己的工作对他人的生活发挥了积极的作用 |  |  |  |  |  |  |
| 10 | 自从从事了这份工作后，我对人越来越冷漠了 |  |  |  |  |  |  |
| 11 | 我担心这份工作会使我变成一个硬心肠的人 |  |  |  |  |  |  |
| 12 | 我觉得自己精力充沛 |  |  |  |  |  |  |
| 13 | 我感到我在工作中受到挫折 |  |  |  |  |  |  |
| 14 | 我感到自己的工作过于辛苦 |  |  |  |  |  |  |
| 15 | 我不太重视病人提出的各种需求 |  |  |  |  |  |  |
| 16 | 与其他人一起工作使我感到有很大的压力 |  |  |  |  |  |  |
| 17 | 与病人一起时 我可以轻易地营造轻松的气氛 |  |  |  |  |  |  |
| 18 | 工作中与病人密切的接触，使我感到满足和愉快 |  |  |  |  |  |  |
| 19 | 从事护理工作使我体验到自身的价值所在 |  |  |  |  |  |  |
| 20 | 我感到自己的情感到了山穷水尽的地步 |  |  |  |  |  |  |
| 21 | 我能冷静的处理工作中遇到的情绪困扰 |  |  |  |  |  |  |
| 22 | 我觉得某些病人把他们应面对的问题归咎于我 |  |  |  |  |  |  |
